# What the papers say

**DOI:** 10.1093/jhps/hnaf053

**Published:** 2025-09-25

**Authors:** Ali Bajwa

**Affiliations:** Orthiopaedics, The Villar Bajwa Practice, Spire Cambridge Lea Hospital, 30 New Road, Impington, Cambridge CB24 9EL, United Kingdom

**Keywords:** Hip, arthroscopy, update, labral tears, college athletes, osteotomy, lateral hip instability

## Abstract

The Journal of Hip Preservation Surgery (JHPS) is not the only place where work in the field of hip preservation can be published. Although our aim is to offer the best of the best, we are continually fascinated by work, that finds its way into journals other than our own. There is much to learn from it, and so JHPS has selected six recent and topical subjects for those who seek a summary of what is taking place in our ever-fascinating world of hip preservation. What you see here are the mildly edited abstracts of the original articles, to give them what JHPS hopes is a more readable feel. If you are pushed for time, what follows should take you no more than 10 min to read. So here goes …

## BIOMETRIC CHANGES UP TO 1 YEAR AFTER BILATERAL HIP ARTHROSCOPY IN DIVISION I COLLEGIATE ATHLETES

The authors from the USA [1] note that patients undergoing bilateral hip arthroscopy (HAS) for femoroacetabular impingement (FAI) require weight-bearing restrictions that, combined with surgery, may influence muscle mass and bone mineral density (BMD).

Their hypothesis was that after bilateral HAS, patients will exhibit changes in biometric data.

In this retrospective cohort study National Collegiate Athletic Association (NCAA) Division I collegiate athletes undergoing bilateral HAS surgery had a dual energy x-ray absorptiometry scan preoperatively and at 3, 6, or 12 months postoperatively. Linear mixed effects models assessed change in total body and leg lean and fat mass and pelvic, spine, and total leg BMD at each timepoint relative to baseline.

Significant decreases (*n* = 18 athletes) in pelvic BMD were seen at baseline [1.40 g/cm^2^; (95% CI, 1.30, 1.51)]; 3 months [1.33 g/cm^2^ (1.23, 1.43)], and 6 months [1.33 g/cm^2^ (1.22, 1.43)], and spine BMD at 6 months postoperatively [1.43 g/cm^2^ (1.30, 1.52); 1.38 g/cm^2^; (1.29, 1.47)]. Lower extremity and total body fat mass were increased at 3 months [6881 g; (4932, 8829); 7757 g (5801, 9713)], [19 565 g; (13 130, 25 999); 21 467 g; (15 012, 27 922)], whereas total body lean mass was decreased at 3 months [66 736 g; (58 265, 75 207); 64 978 g; (56 492, 73 464)]. Results at 12 months were not significantly different from baseline.

The authors concluded that in NCAA Division I collegiate athletes who underwent bilateral HAS for FAI, pelvic and spine BMD was decreased up to 6 months after surgery when compared with presurgery measurements, but no decrease was observed after 1 year. Clinicians should be aware of the potential implications of decreased bone mass up to 12 months postsurgery. Understanding changes in body composition and BMD postoperatively may help to guide rehabilitation management and injury risk assessment.

## SIX-MONTH FUNCTIONAL SCORES PREDICT 5-YEAR ACHIEVEMENT OF THE MINIMAL CLINICALLY IMPORTANT DIFFERENCES AFTER HIP ARTHROSCOPY FOR SYMPTOMATIC ACETABULAR LABRAL TEARS

In this study, Gillinov *et al*. [2] note that despite the increasing utilization of HAS and identification of predictors of poor outcomes, the effect of short-term improvement on long-term functional outcomes has been understudied. The purpose of their study was to determine whether improvements in patient-reported outcomes (PROs) 6 months after HAS predict 5-year outcomes.

In this case–control study, a retrospective review of prospectively collected data identified patients ≥18 years who underwent primary HAS by a single surgeon for the treatment of symptomatic labral tears. Included patients had a Tönnis grade < 2 and completed PROs at baseline, 6-month, and minimum 2-year follow-up, and annually thereafter. The minimal clinically important difference (MCID) for the modified Harris Hip Score (mHHS), 8 points, was used to stratify patients into cohorts based on high improvement (HI) versus low improvement (LI) at 6 months. PROs were compared at 1, 2, 3, 4, and 5 years postoperatively by rates of MCID achievement and linear mixed-effects modeling. Subsequent surgery rates were compared by chi-square or Fisher exact tests, as appropriate.

Overall, 175 patients (age, 37.2 ± 11.4 years; 52.0% female) met inclusion criteria. Of these, 131 HI patients were compared with 44 LI patients. At 5 years, 88.3% of HI patients reached MCID, versus 42.1% of LI patients. By multivariable logistic regression, achievement of 6-month MCID [adjusted odds ratio (AOR), 17.43] and labral management (augmentation, relative to debridement: AOR, 14.5) predicted achievement of 5-year MCID. mHHS scores were greater for HI versus LI patients through 3-year follow-up but were not significantly different at 4 and 5 years. Subsequent surgery rates were 9.9% and 11.4% in HI versus LI patients, respectively.

In conclusion the study demonstrates that early functional improvements after HAS, assessed by 6-month MCID, predicted clinically meaningful outcomes at 5-year follow-up, underscoring the importance of early cautious recovery to prioritize labral healing while also meeting appropriate, stepwise rehabilitation milestones to advance functionally during these 6 months. Despite this, LI patients continued improving for 5 years, demonstrating that late functional improvements are still possible for certain patients in the event of a poor 6-month rehabilitation period.

## PERIACETABULAR OSTEOTOMY VERSUS HIP ARTHROSCOPY IN PATIENTS WITH BORDERLINE DEVELOPMENTAL DYSPLASIA OF THE HIP: A SYSTEMATIC REVIEW AND MULTI-LEVEL META-ANALYSIS

The authors from Germany [3] state that a comprehensive meta-analysis is required to address the lack of quantitative evidence on treatment outcomes in borderline developmental dysplasia of the hip (BDDH). Their study compares periacetabular osteotomy (PAO) and HAS through a multi-level meta-analysis, providing quantitative insights into their efficacy and safety.

A systematic literature search was conducted in PubMed, Epistemonikos, and Embase up to 28 February 2025. A frequentist meta-analysis was performed using the Hartung-Knapp-Sidik-Jonkman heterogeneity estimator. Continuous variables were analyzed using mean values with 95% confidence intervals (CIs), and binary outcomes as proportions with 95% CIs. Sensitivity analysis compared studies defining BDDH as 20°–25° versus all included studies. Statistical heterogeneity was assessed using Higgins' I^2^. A random-effects model was applied in cases of significant heterogeneity.

The literature search identified 39 primary studies with a total of 2075 patients (2121 hips). The test for subgroup differences showed no statistically significant difference between the PAO group and the HAS group in postoperative mHHS (*χ*^2^ = 0.55; df = 1; *P* = .46), in postoperative iHOT-12 (*χ*^2^ = 0.00; df = 1; *P* = .98), in the change in mHHS (*χ*^2^ = 0.37; df = 1; *P* = .54), in the change in iHOT-12 (*χ*^2^ = 1.05; df = 1; *P* = .30), in MCID of postoperative functional outcome scores (*χ*^2^ = 0.43; df = 1; *P* = .51), in reoperation (*χ*^2^ = 0.17; df = 1; *P* = .68), and in complications (*χ*^2^ = 3.35; df = 1; *P* = .07). The absolute mean values for nearly all parameters favor PAO, which may indicate a potential advantage.

The authors thus concluded that PAO and HAS yield comparable short- to mid-term outcomes in BDDH. Long-term studies are needed to determine whether HAS is a definitive treatment or delays structural correction. Future research should standardize BDDH definitions (LCEA 20°–25°) to enhance comparability and treatment consistency.

## THE LATERAL HIP INSTABILITY TEST: DIAGNOSTIC ACCURACY FOR LATERAL OR POSTEROLATERAL FEMORAL HEAD UNDERCOVERAGE

In the study from Germany, Wettstein *et al*. [4] aim to (i) describe the lateral hip instability test, developed to discriminate between stable versus unstable hips with lateral or posterolateral femoral head undercoverage, (ii) evaluate differences between painful hips that tested positive versus negative, and (iii) evaluate the accuracy of this test as defined by radiographic references for acetabular dysplasia and/or retroversion.

A consecutive series of patients were evaluated for hip pain from 1 January 2019 to 31 January 2021. Routine assessment included the new lateral hip instability test, which is positive when inducing deep lateral hip pain and consists of maximum passive adduction of the painful hip, and application of a force in the long axis of the femur. Descriptive statistics were used to compare patients with positive versus negative lateral hip instability tests. The accuracy of the test was assessed using a lateral center-edge angle (LCEA) ≤ 20° for frank dysplasia, LCEA ≤ 25° for borderline dysplasia, and a combination of positive crossover sign, posterior wall sign, and ischial spine sign for acetabular retroversion.

Sixty-nine of the 159 (43%) painful hips had a positive lateral hip instability test. Positive hips had higher Tönnis angles (9° ± 8° versus 3° ± 5°), lower LCEA (22° ± 8° versus 29° ± 6°), and greater proportions of frank dysplasia (49% versus 6%), borderline dysplasia (70% versus 24%) and acetabular retroversion (52% versus 27%). The lateral hip instability test had a sensitivity of 63% and a specificity of 96% for detecting borderline dysplasia and/or acetabular retroversion, a sensitivity of 68% and a specificity of 87% for detecting frank dysplasia and/or acetabular retroversion.

The authors concluded that the lateral hip instability test can discriminate between stable versus unstable painful hips with lateral and/or posterolateral femoral head undercoverage. When the test is positive, clinicians can be quite certain that a patient has hip instability resulting from lateral and/or posterolateral undercoverage.

## HEATED IRRIGATION FLUIDS DID NOT REDUCE THE PREVALENCE OF RECTALLY MEASURED HYPOTHERMIA DURING HIP ARTHROSCOPIC SURGERY COMPARED WITH ROOM-TEMPERATURE FLUIDS: A PROSPECTIVE RANDOMIZED TRIAL

The authors from Turkey [5] explore an important question in HAS. They note that close monitoring and heated irrigation fluids have been frequently used to avoid hypothermia and associated complications during hip arthroscopic surgery. Saline fluids are used extensively in hip arthroscopic surgery, but they are routinely stored at room temperature and are cooler than the patient's core temperature.

The purpose of the study was to investigate the efficacy of heated irrigation fluids to prevent hypothermia during hip arthroscopic surgery and whether the core temperature measured rectally during hip arthroscopic surgery differs from the core temperature measured at the temporal region in a randomized controlled trial.

Patients who underwent hip arthroscopic surgery for the treatment of FAI syndrome between 2021 and 2023 were prospectively enrolled and divided into 2 groups: those whose irrigation fluids were stored at room temperature (group 1) and those whose irrigation fluids were heated to 36°C to 38°C (group 2). A probe inserted in the rectal mucosa was used to measure the patient's body temperature every 15 min. The patient’s temperature was also measured at the temporal region with a laser thermometer. A body temperature < 36°C, detected by either method, was considered as hypothermia. The method that detected hypothermia more quickly was investigated, and the effect of the heated irrigation fluids was explored. Statistical analyses were conducted to compare temperature measurements and the incidence of hypothermia between the groups using appropriate tests for categorical and continuous variables based on the data distribution.

There were 60 patients randomized and allocated to group 1 and 56 patients to group 2. Hypothermia, defined as a temperature < 36°C, occurred in 32 patients (53.3%) in group 1 and 24 patients (42.9%) in group 2. There was no difference between the groups using heated or room-temperature fluids in the onset of hypothermia. Significantly more hypothermia cases were detected by the rectal temperature measurement than by the temporal temperature measurement (54 versus 2 patients, respectively). The rectal temperature measurement was also quicker in detecting hypothermia (69.6 ± 47.2 versus 138.2 ± 56.8 min, respectively).

This study demonstrates that the usage of either room-temperature or heated irrigation fluids did not influence the incidence of hypothermia. Rectal measurements of core body temperature detected hypothermia earlier during hip arthroscopic surgery.

## INCREASED LENGTH OF SURGERY IS ASSOCIATED WITH INCREASED FLUID PUMP PRESSURE DURING HIP ARTHROSCOPY

In this study, Felan *et al*. [6] state that elevated fluid pump pressures during HAS have been associated with surgical complications, including fluid extravasation, abdominal compartment syndrome, and higher use of pain medication.

The purpose of the study was to demonstrate the safe use of low pump pressures and to identify factors associated with the need for increased fluid pump pressure during HAS.

In this case–control study, a retrospective review was performed on all patients who underwent HAS by a single surgeon between 2020 and 2021. Patients maintaining a pump pressure ≤ 20 mm Hg during surgery were assigned to group A, and patients who required a pump pressure > 20 mm Hg at any point during surgery were assigned to group B. Univariate and multivariate analyses were performed to assess for relationships between elevated pump pressure (>20 mm Hg) during HAS and the patient’s age, sex, length of surgery, body mass index, Beighton hypermobility score, lateral center edge angle, alpha angle, Tegner activity score, femoral shaft torsion angle, highest systolic blood pressure during surgery, number of suture anchors, and the presence of medical comorbidities (i.e. hypertension, clotting disorder, or diabetes mellitus).

A total of 103 patients (112 hips) were included. Group A consisted of 77 hips (54 female, 23 male) with a mean age of 33.3 years. Group B consisted of 35 hips (19 female, 16 male) with a mean age of 33.6 years. Multivariate regression analysis revealed that mean length of surgery was associated with a need to increase pump pressure (*P* < .001). On average, for every 1-min increase in surgery duration, the odds of needing an increase in pump pressure increased by 2.0%. In addition, the highest systolic blood pressure during surgery and the number of anchors were associated with an elevated pump pressure on univariate analysis.

The authors thus concluded that for a majority of patients who underwent HAS, a pump pressure of 20 mm Hg resulted in adequate visualization for the duration of the case. Increased length of surgery was associated with an elevated pump pressure during HAS.

Conflict of interest: None declared.
